# Quantitative radiomic profiling of glioblastoma represents transcriptomic expression

**DOI:** 10.18632/oncotarget.23975

**Published:** 2018-01-05

**Authors:** Doo-Sik Kong, Junhyung Kim, Gyuha Ryu, Hye-Jin You, Joon Kyung Sung, Yong Hee Han, Hye-Mi Shin, In-Hee Lee, Sung-Tae Kim, Chul-Kee Park, Seung Hong Choi, Jeong Won Choi, Ho Jun Seol, Jung-Il Lee, Do-Hyun Nam

**Affiliations:** ^1^ Department of Neurosurgery, Samsung Medical Center, Sungkyunkwan University, Seoul, Republic of Korea; ^2^ Institute for Refractory Cancer Research, Samsung Medical Center, Seoul, Republic of Korea; ^3^ Department of Health Science and Technology, Samsung Advanced Institute of Health Science and Technology, Sungkyunkwan University, Seoul, Republic of Korea; ^4^ Department of Computer Science, Korea University, Seoul, Republic of Korea; ^5^ Medical System Research Department, Convergence Technology Institute, Hyundai Heavy Industries, Co., Ltd, Ulsan, Republic of Korea; ^6^ Department of Radiology, Samsung Medical Center, Seoul, Republic of Korea; ^7^ Department of Neurosurgery, Seoul National University Hospital, Seoul, Republic of Korea; ^8^ Department of Radiology, Seoul National University Hospital, Seoul, Republic of Korea

**Keywords:** quantitative imaging, glioblastoma, radiomic, classification, phenotypes

## Abstract

Quantitative imaging biomarkers have increasingly emerged in the field of research utilizing available imaging modalities. We aimed to identify good surrogate radiomic features that can represent genetic changes of tumors, thereby establishing noninvasive means for predicting treatment outcome. From May 2012 to June 2014, we retrospectively identified 65 patients with treatment-naïve glioblastoma with available clinical information from the Samsung Medical Center data registry. Preoperative MR imaging data were obtained for all 65 patients with primary glioblastoma. A total of 82 imaging features including first-order statistics, volume, and size features, were semi-automatically extracted from structural and physiologic images such as apparent diffusion coefficient and perfusion images. Using commercially available software, NordicICE, we performed quantitative imaging analysis and collected the dataset composed of radiophenotypic parameters. Unsupervised clustering methods revealed that the radiophenotypic dataset was composed of three clusters. Each cluster represented a distinct molecular classification of glioblastoma; classical type, proneural and neural types, and mesenchymal type. These clusters also reflected differential clinical outcomes. We found that extracted imaging signatures does not represent copy number variation and somatic mutation. Quantitative radiomic features provide a potential evidence to predict molecular phenotype and treatment outcome. Radiomic profiles represents transcriptomic phenotypes more well.

## INTRODUCTION

Glioblastoma (GBM) is the most common and fatal type of glioma, accounting for 40% of all malignant primary brain tumors and showing a median survival of 14 months despite treatment by surgical resection followed by concurrent chemoradiotherapy. This dismal prognosis is largely attributed to the cancer heterogeneity of GBM [1]. Thus, great effort has been made to advance technology in the fields of molecular and genomic biology in order to better characterize individual cancer.

Quantitative magnetic resonance (MR) imaging can be defined as the extraction of quantifiable features from advanced magnetic resonance images and quantitatively describes the tumor phenotypes [2, 3]. At the initial diagnosis or during treatment, quantitative imaging biomarkers can represent a biomarker of normal biological or pathological processes or treatment response to therapeutics [2]. To date, MR-based morphological measurements using T1 contrast-enhancement or T2 FLAIR imaging are well-known and widely accepted quantitative methods under some clinical situations such as Alzheimer disease, osteoarthritis, and a variety of cancers [2]. Several studies have demonstrated that MR imaging signatures can indicate clinical outcomes in high-grade gliomas [4]. However, few studies have demonstrated that physiologic biomarkers derived from conventional imaging data are closely associated with clinical outcome or have investigated their association with the underlying genetic messages [5]. Recently, rapid developments in advanced imaging analysis for tumor classification or quantification of tumor vascularity and permeability have been introduced. In radiographic studies of glioblastoma, physiologic imaging biomarkers are gradually being uncovered and are believed to be crucial for predicting the treatment response of the tumor or natural course of tumor progression.

Here, we report a comprehensive radiogenomic study of glioblastoma based on integration of quantitative large-scale radiomic profiling and genomic data. We integrated structural and physiologic MR imaging data with multi-platform genomic data from 65 glioblastomas. We also evaluated quantitative large-scale radiomic profiling to identify statistically significant associations between genomic features and radiomic signatures in glioblastomas.

## RESULTS

### Patient characteristics

From May 2012 to June 2014, we retrospectively identified 65 patients with treatment-naïve GBM with available clinical information from the Samsung Medical Center data registry. The median age of the patients was 58.7 years (range, 29-74 years), and the population was composed of 35 males and 30 females. 46 of 65 patients (70.8%) were expired at the investigation and median overall survival of the total population was 13.2 months (95% CI 9.3-17.6 months). The metadata for the 65 GBM samples are provided in Table 1.

**Table 1:** The parameters used in this study

Feature	No.	Parameters	Image sequence	Definitions
Volume	1	T1CE_PIXEL	T1CE	Number of pixel intensity based on ROI of T1CE
	2	T1CE_AREA	T1CE	Mask area (cm^2^) based on ROI of T1CE
	3	T1CE_VOL	T1CE	Mask volume (mL) based on ROI of T1CE
	4	T2FLAIR_PIXEL	T2FLAIR	Number of pixel intensity on ROI of T2FLAIR
	5	T2FLAIR_AREA	T2FLAIR	Mask area (cm^2^) based on ROI of T2FLAIR
	6	T2FALIR_VOL	T2FLAIR	Mask volume (mL) based on ROI of T2FLAIR
	7	rADCnT1_PIXEL	rADC	Number of pixel intensity on T1CE ROI
	8	rADCnT1_AREA	rADC	Mask area (cm^2^) on T1CE ROI
	9	rADCnT1_VOL	rADC	Mask vol (mL) on T1CE ROI
	10	rADCnT2_PIXEL	rADC	Number of pixel intensity on T2FLAIR ROI
	11	rADCnT2_AREA	rADC	Mask area (cm^2^) on T2FLAIR ROI
		rADCnT2_VOL	rADC	Mask vol (mL) on T2FLAIR ROI
Cerebral blood flow	13	rCBFnT1_MEAN	rCBF	Mean based on ROI of T1CE
	14	rCBFnT1_MEDIAN	rCBF	Median based on ROI of T1CE
	15	rCBFnT1_SD	rCBF	SD based on ROI of T1CE
	16	rCBFnT1_X5P	rCBF	5 percentile of total intensity on ROI of T1CE
	17	rCBFnT1_X95P	rCBF	95 percentile of total intensity on ROI of T1CE
	18	rCBFnT1_Q1	rCBF	25 percentile of total intensity on ROI of T1CE
	19	rCBFnT1_Q3	rCBF	75 percentile of total intensity on ROI of T1CE
	20	rCBFnT2_MEAN	rCBF	Mean based on ROI of T2FLAIR
	21	rCBFnT2_MEDIAN	rCBF	Median based on ROI of T2FLAIR
	22	rCBFnT2_SD	rCBF	SD based on ROI of T2FLAIR
	23	rCBFnT2_X5P	rCBF	5 percentile of total intensity on ROI of T2FLAIR
	24	rCBFnT2_X95P	rCBF	95 percentile of total intensity on ROI of T2FLAIR
	25	rCBFnT2_Q1	rCBF	25 percentile of total intensity on ROI of T2FLAIR
	26	rCBFnT2_Q3	rCBF	75 percentile of total intensity on ROI of T2FLAIR
Cerebral blood volume	27	rCBVnT1_MEAN	rCBV	Mean based on ROI of T1CE
	28	rCBVnT1_MEDIAN	rCBV	Median based on ROI of T1CE
	29	rCBVnT1_SD	rCBV	SD based on ROI of T1CE
	30	rCBVnT1_X5P	rCBV	5 percentile of total intensity on ROI of T1CE
	31	rCBVnT1_X95P	rCBV	95 percentile of total intensity on ROI of T1CE
	32	rCBVnT1_Q1	rCBV	25 percentile of total intensity on ROI of T1CE
	33	rCBVnT1_Q3	rCBV	75 percentile of total intensity based on ROI of T1CE
	34	rCBVnT2_MEAN	rCBV	Mean based on ROI of T2FLAIR
	35	rCBVnT2_MEDIAN	rCBV	Median based on ROI of T2FLAIR
	36	rCBVnT2_SD	rCBV	SD based on ROI of T2FLAIR
	37	rCBVnT2_X5P	rCBV	5 percentile of total intensity on ROI of T2FLAIR
	38	rCBVnT2_X95P	rCBV	95 percentile of total intensity on ROI of T2FLAIR
	39	rCBVnT2_Q1	rCBV	25 percentile of total intensity on ROI of T2FLAIR
	40	rCBVnT2_Q3	rCBV	75 percentile of total intensity on ROI of T2FLAIR
Mean transit time	41	MTTnT1_MEAN	MTT	Mean based on ROI of T1CE
	42	MTTnT1_MEDIAN	MTT	Median based on ROI of T1CE
	43	MTTnT1_SD	MTT	SD based on ROI of T1CE
	44	MTTnT1_X5P	MTT	5 percentile of total intensity based on ROI of T1CE
	45	MTTnT1_X95P	MTT	95 percentile of total intensity based on ROI of T1CE
	46	MTTnT1_Q1	MTT	25 percentile of total intensity based on ROI of T1CE
	47	MTTnT1_Q3	MTT	75 percentile of total intensity based on ROI of T1CE
	48	MTTnT2_MEAN	MTT	Mean based on ROI of T2FLAIR
	49	MTTnT2_MEDIAN	MTT	Median based on ROI of T2FLAIR
	50	MTTnT2_SD	MTT	SD based on ROI of T2FLAIR
	51	MTTnT2_X5P	MTT	5 percentile of total intensity on ROI of T2FLAIR
	52	MTTnT2_X95P	MTT	95 percentile of total intensity on ROI of T2FLAIR
	53	MTTnT2_Q1	MTT	25 percentile of total intensity on ROI of T2FLAIR
	54	MTTnT2_Q3	MTT	75 percentile of total intensity on ROI of T2FLAIR
Time to perfusion	55	TTPnT1_MEAN	TTP	Mean based on ROI of T1CE
	56	TTPnT1_MEDIAN	TTP	Median based on ROI of T1CE
	57	TTPnT1_SD	TTP	SD based on ROI of T1CE
	58	TTPnT1_X5P	TTP	5 percentile of total intensity based on ROI of T1CE
	59	TTPnT1_X95P	TTP	95 percentile of total intensity based on ROI of T1CE
	60	TTPnT1_Q1	TTP	25 percentile of total intensity based on ROI of T1CE
	61	TTPnT1_Q3	TTP	75 percentile of total intensity based on ROI of T1CE
	62	TTPnT2_MEAN	TTP	Mean based on ROI of T2FLAIR
	63	TTPnT2_MEDIAN	TTP	Median based on ROI of T2FLAIR
	64	TTPnT2_SD	TTP	SD based on ROI of T2FLAIR
	65	TTPnT2_X5P	TTP	5 percentile of total intensity on ROI of T2FLAIR
	66	TTPnT2_X95P	TTP	95 percentile of total intensity on ROI of T2FLAIR
	67	TTPnT2_Q1	TTP	25 percentile of total intensity on ROI of T2FLAIR
	68	TTPnT2_Q3	TTP	75 percentile of total intensity on ROI of T2FLAIR
Apparent diffusion coefficient	69	rADCnT1_MEAN	rADC	Mean based on ROI of T1CE
	70	rADCnT1_MEDIAN	rADC	Median based on ROI of T1CE
	71	rADCnT1_SD	rADC	SD based on ROI of T1CE
	72	rADCnT1_X5P	rADC	5 percentile of total intensity based on ROI of T1CE
	73	rADCnT1_X95P	rADC	95 percentile of total intensity based on ROI of T1CE
	74	rADCnT1_Q1	rADC	25 percentile of total intensity based on ROI of T1CE
	75	rADCnT1_Q3	rADC	75 percentile of total intensity based on ROI of T1CE
	76	rADCnT2_MEAN	rADC	Mean based on ROI of T2FLAIR
	77	rADCnT2_MEDIAN	rADC	Median based on ROI of T2FLAIR
	78	rADCnT2_SD	rADC	SD based on ROI of T2FLAIR
	79	rADCnT2_X5P	rADC	5 percentile of total intensity on ROI of T2FLAIR
	80	rADCnT2_X95P	rADC	95 percentile of total intensity on ROI of T2FLAIR
	81	rADCnT2_Q1	rADC	25 percentile of total intensity on ROI of T2FLAIR
	82	rADCnT2_Q3	rADC	75 percentile of total intensity on ROI of T2FLAIR

### Consensus clustering identifies three subtypes of GBM

**Figure 1:**
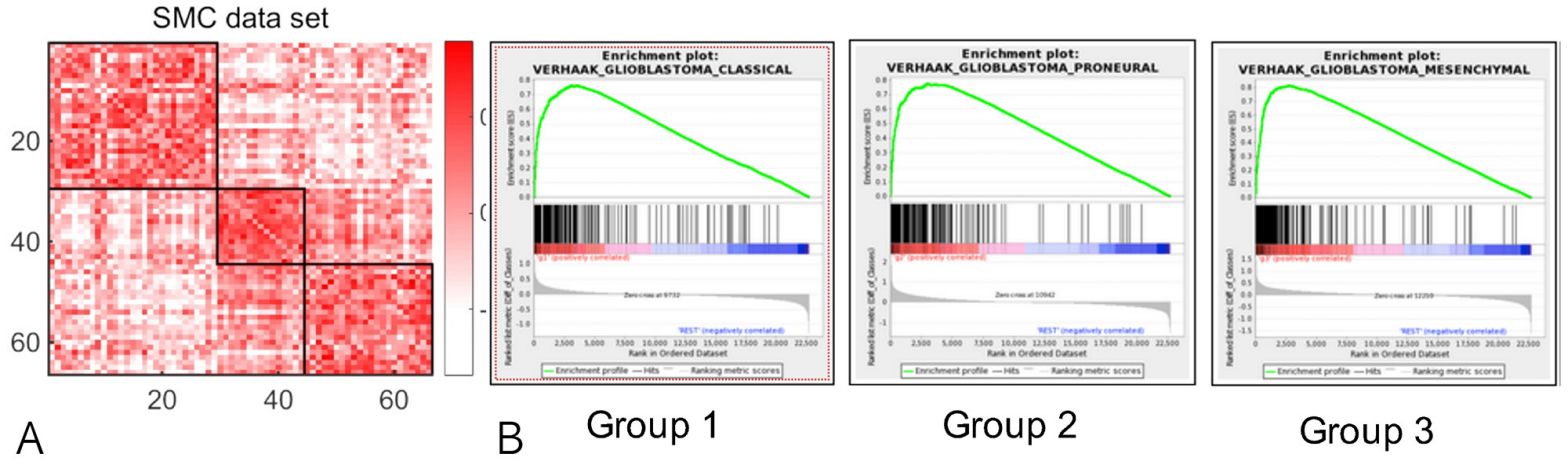
Three radiomic clusters **(A)** Similarity matrix based on Pearson’s correlation coefficients among 65 glioblastoma cases. **(B)** Gene-set enrichment analysis (GSEA) results for each radiomic cluster. Identified associations are enriched for certain categories of genomic features and radiomic phenotypes, evaluated by the adjusted p-values from the Fisher’s exact tests.

We performed a quantitative study to investigate the association of genomic features such as gene expression or copy number variation with 82 radiomic phenotypes (Table 1). Table 1 summarizes the name and brief description of all 82 radiomic profiles. A total of 5,330 quantitative MR imaging features (representing first-order statistics, size, and volume) were extracted from volume masks to maximize characterization of the tumor (82 x 65, no of features x no of patients). Using a high-throughput approach, we identified radiomic signatures consisting of 82 MR imaging features that segregated patients with GBM into three distinct consensus clusters (Figure 1A). The similarity matrix formed by Pearson’s correlation coefficients of the 65 samples suggested three robust stable clusters. Based on the gene functions of discriminatory mRNA transcripts, radiomic clustering represented interpretation in the context of existing molecular subclassification of GBM.

### Association between genetic pathway and radiomic phenotypes

For each of the 82 radiomic phenotypes, we investigated the Gene Set Enrichment Analysis (GSEA) for identifying the KEGG pathways whose transcriptional changes associated with the change of tumor radiomic phenotype. The gene-level statistics used to characterize the association between gene expression and radiomic phenotype were obtained by the p-value resulted from the Spearman rank correlation test. ClaNC, a nearest centroid-based classifier that balances the number of genes per class, identified signature genes for all three subtypes. An 840 gene signature (210 genes per class), was established from the smallest gene set with the lowest cross validation (CV) and prediction error. As a proof of concept, we showed that a significant number of genes overexpressed in radiomic subgroup 1 were associated with anion channel activity, peroxisome, and the classic subtype of GBM suggested by Verhaak et al. [1]. Group 2 was characterized by high expression of genes associated with mitosis, cell cycle, ribosomes, and the proneural or neural subtype. The third group showed overexpression of extracellular matrix molecules, defense response, immune signaling molecules, and the mesenchymal subtype (Figure 1B).

### Sample-specific differentially expressed genes

**Figure 2:**
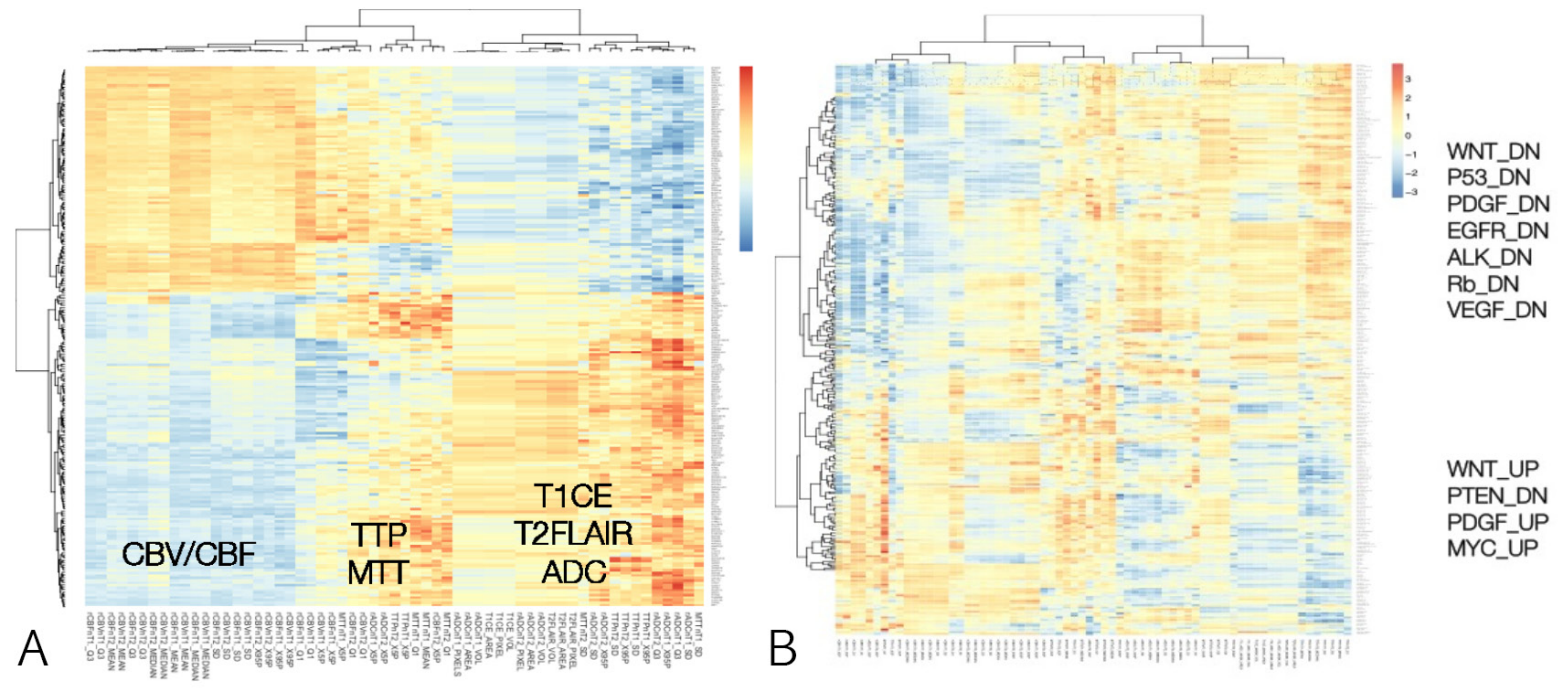
Overview of identified statistically significant associations **(A)** Heatmap of RNA sequencing data demonstrating correlations between transcriptomic profile and radiomic signatures. **(B)** Correlation matrix between radiomic data and gene expression profiles are plotted in the heatmap. Associations were deemed as statistically significant if the adjusted p-value ≤ 0.01.

We investigated the correlation matrix between radiomic data and gene expression profiles in the heatmap (Figure 2A). We found that each radiomic profile representing volume, blood flow or volume and diffusion had his own molecular characteristics. To identify pathways that are differentially correlated with radiomic phenotypes, using these four gene sets, a single-sample GSEA (ssGSEA) enrichment score was calculated for all samples. We used ssGSEA projection as a hypothesis-generating gene set identification tool. Single sample GSEA identifies biologically relevant gene sets that correlate with a radiomic phenotypes by estimating pathway activities of the radiomic phenotypes in this study. We performed an unsupervised clustering analysis to associate transcriptional activities of all genetic pathways in the Kyoto Encyclopedia of Genes and Genomes (KEGG) database with radiomic phenotypes (Figure 2B). As a result, we found each physiologic or structural MR imaging features showed its own transcriptomic pathway. Specifically, pathway transcriptional activities were associated with radiomic phenotypes with statistical significance. Radiomic features related to CBV and CBF showed upregulation of Wnt, PDGF, EGFR, ALK, Rb, VEGF, and Myc pathways and downregulation of PTEN pathway (p < 0.01). In contrast, radiomic features representing volumetric parameters based on T1CE, T2FLAIR, or ADC demonstrated an inverse relationship with radiomic features representing CBV or CBF.

### Associations between somatic gene mutations/ CNV and radiomic phenotypes

**Figure 3:**
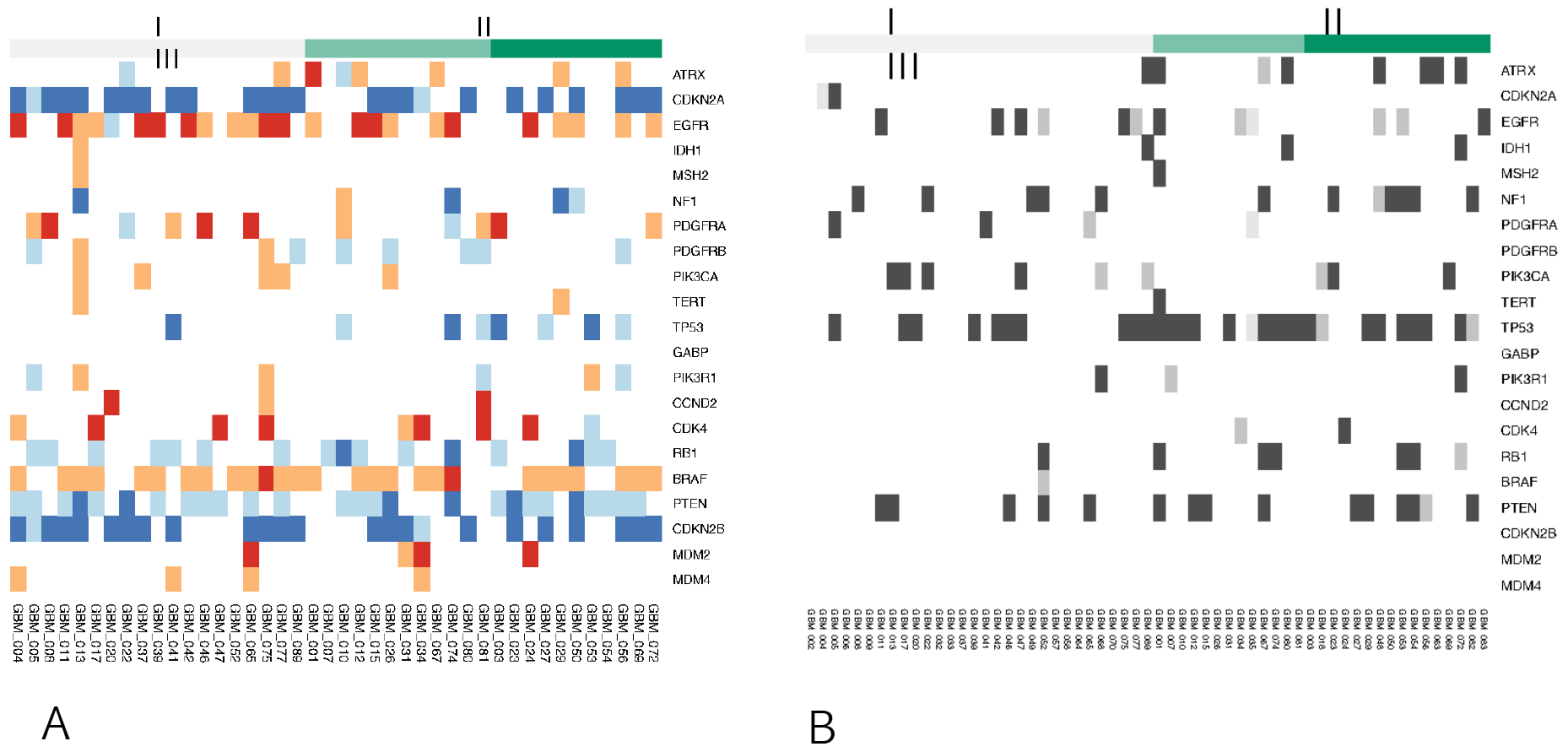
Genetic profiles of each radiomic cluster for representative genes I means group 1, II means group 2, and III means group 3. The number of patients with exactly the same mutation and CNV was quite small and might not provide reliable statistics. **(A)** Copy number alterations (log2CN). **(B)** Somatic mutations.

We compared measurements of a radiomic phenotype for patients harboring somatic mutations in a gene versus those without. For each mutation/ CNV and each radiomic phenotype, a linear regression was used to fit the value of the radiomic phenotype and examine whether the mutation has a significant effect on the phenotype. However, the number of patients with exactly the same mutation was quite small and might not provide reliable statistics. Compared to the transcriptional activities of genetic pathways, the CNVs and representative somatic mutations showed far fewer statistically significant associations (Figure 3).

## DISCUSSION

Radiogenomics investigates the association between imaging phenotypes and genomic features and is an emerging field in cancer medicine. As an inexpensive and noninvasive method, quantitative radiomics can provide the great promise of personalized medicine by inferring underlying genomic phenotypes. It can also serve as an imaging biomarker to characterize the underlying genomics. If the radiomic data can represent the temporal-spatial intra/inter tumoral heterogeneity, repeated MR imaging would be a powerful tool to show the molecular status of a tumor sample using a non-invasive technique [9]. In the field of radiologic studies of glioblastomas, physiologic MR imaging and structural MR imaging provide invaluable information about the tumor characteristics. For instance, dynamic susceptibility contrast (DSC) perfusion MR imaging is commonly used in clinical practice to interpret the tumor microvasculature for the purpose of estimation of rCBV or MTT for cerebral blood flow or volume. In addition, apparent diffusion coefficient (ADC) imaging is another important MR imaging biomarker that represents the cellular density and the movement of free fluid water. Interpretation of advanced physiologic imaging requires specialized well-trained radiologists; however, interpretation is still subjective and can produce potential inter-observer variability. To overcome this drawback, attempts to quantify the imaging data have aimed to identify and characterize imaging biomarkers, but prediction of the genomic characteristics remains challenging.

To date, several studies have reported that imaging features represent the clinical outcome in patients with malignant glioma [4, 9–17]. Most studies have investigated clinical application in glioblastomas by quantification of structural imaging such as volume or shape [13, 15, 18–23], while comprehensive integration of physiologic imaging such as diffusion or perfusion imaging, clinical outcome, and genomic profiles has been rarely reported [4, 9, 16, 24, 25]. Integrated analyses using gene expression, copy number, methylation, and somatic mutation patterns have demonstrated distinct GBM subtypes, possibly related to the treatment response and clinical outcome [1, 26]. Several authors have demonstrated that specific molecular subtypes in glioblastoma correlate with certain imaging traits [22, 27]. We present a comprehensive radiogenomic study of glioblastoma based on the integration of multi-omics molecular data from our genome data bank with MRI-based radiomic data for 65 glioblastomas. We found that radiomic data could provide information about molecular subtypes of GBM based on the transcriptomic profile. In addition, the radiomic group associated with mesenchymal subtypes showed a worse prognosis than other groups. Compared with the close relationship between transcription level and radiomic phenotype, we identified far fewer statistically significant associations for CNVs and gene somatic mutations. This finding was compatible with the results of a breast cancer study by Zhu et al. [28]. Genetic mutations are further upstream in the functional activities of the cellular system, and Zhu et al. suggested that gene expressions are therefore more closely associated with phenotype in the process of genetic events influencing phenotype development. On unsupervised clustering analysis between radiomic features and transcriptomic profiles, multiple parameters representing CBV and CBF had similar transcriptomic patterns and parameters, indicating that MTT and TTP have their own genomic signatures. Volumetric radiomic data had a similar genomic pattern to ADC. This finding demonstrates that specific radiomic phenotypes have similar genomic signature patterns.

However, our study has some limitations for interpreting the association between genomic signature and imaging phenotype. Genomic data in this study were generated using a single tissue sample from each primary tumor. A single biopsy sample of a tumor cannot represent a heterogeneous cell population; therefore, the genomic data of a single sample only partially reflects the overall genomic landscape of the entire tumor. In the future, topographic analysis of radiomic data combined with multiple biopsies will provide more intensive and accurate information. Through the study of radiogenomics, we aimed to identify good surrogate radiomic features that can reveal genetic changes of tumors, thereby establishing noninvasive means for monitoring tumor progression. We believe that our initial results provide the motivation to investigate the relationships between the multi-layer molecular system of the tumor and the various quantitative radiomic phenotypes of glioblastoma.

## MATERIALS AND METHODS

### Patient enrollment

Between May 2012 to June 2014, we retrospectively identified 65 patients who met the following criteria: (1) previously untreated and histologically confirmed grade IV GBM according to the World Health Organization (WHO) classification; (2) available clinical variables including patient demographics; (3) available genomic data such as transcriptome and exome; and (4) available preoperative MR imaging data consisting of pre-contrast axial T1-weighted (T1), post-contrast axial T1-weighted (T1CE), T2-weighted fluid attenuation inversion recovery (T2FLAIR) images, MR-diffusion weighted imaging (DWI) for assessment of apparent diffusion coefficient (ADC), and MR-perfusion weighted imaging (PWI) for assessment of relative cerebral blood flow (rCBF), relative cerebral blood volume (rCBV), mean transit time (MTT), and time to perfusion (TTP). These data were obtained from the medical records at Samsung Medical Center, Seoul, Korea. Patients with recurrent GBM, secondary GBM, grade III glioma, or previous history of treatment were excluded from this study. All tissue samples were collected with written informed consent under a protocol approved by the Institutional Review Board (IRB) of Samsung Medical Center (2010-04-004, Seoul, Korea). All patients were treated by the concomitant chemo-radiotherapy followed by adjuvant Temozolomide 6 cycles. Enrolled patients in this study were not enrolled for any clinical trials.

### MRI data acquisition and preprocessing

All MR imaging was preoperatively conducted on a 3-T scanner, and post-contrast images were acquired 5 minutes after injection of contrast agent. The standard MRI protocol included axial T1-weighted imaging, T2-weighted imaging, fluid-attenuated inversion recovery (FLAIR), perfusion-weighted, and diffusion-weighted MR images. Diffusion-weighted images (DWI) were acquired before injection of contrast and were obtained with TE/TR 80 ms/3 s, section thickness 5 mm with 1 mm intersection gap, matrix size 164 × 162, and FOV 24 cm using monopolar spin-echo echo-planar preparation. Apparent diffusion coefficient (ADC) images were calculated from acquired DWI with b-values of 0 s/mm2 and 1,000 s/mm2, and dynamic susceptibility contrast perfusion-weighted images (PWI) (TR/TE 1720/35 ms, flip angle 40°, section thickness 5 mm, acquisition matrix 128 × 128, 50 volumes, acquisition time 1 minute 30 seconds) were performed. ADC maps were generated using an EWS Workstation (Philips Healthcare), and dynamic susceptibility contrast perfusion images were processed using a dedicated software package (nordicICE; NordicNeuroLab, Bergen, Norway).

All Digital Imaging and Communications in Medicine (DICOM) images were adjusted for spatial smoothing, noise filtering, and motion correction as supported in the dedicated software. Gamma variate fitting was applied to avoid a recirculation effect and dynamic curves were corrected mathematically to reduce contrast agent leakage effects.

### Volumetric segmentation and ROI analysis

For region of interest (ROI) analysis, T1CE and T2FLAIR images were evaluated to define tumor margins by manual segmentation. The image processing software package (nordicICE; NordicNeuroLab, Bergen, Norway) was used for the manual ROI-based image segmentation and volume measurement. In detail, two ROI were obtained from both T1CE- and T2FLAIR-based image in each patient. An ROI was drawn on each slice where the contrast-enhancing area on the T1CE image and high signal intensity area on the T2FLAIR image were visible. Each tumor lesion was segmented by two independent neuro-radiologists (ST Kim and HY Kim) for both T1CE and T2FLAIR modalities. Regions with central necrotic area and normal vascular structures were subtracted from ROIs. Single ROIs from each case were selected for further analysis with consensus between reviewers. Volumes of masked ROIs were also validated with a fully automated method using BraTumIA v1.2 [6], which shows results consistent with previous studies [7, 8]. All MR images were transferred to a high-performance cluster server for post-processing. After post-processing of perfusion and diffusion-weighted images to generate rCBV and ADC maps from both T1CE and T2FLAIR images (5%, 25%, 75%, 95% of total intensity, mean, standard deviation and median value), histogram statistics from voxels in the ROI were extracted. Several volumetric parameters (number of pixels in mask on ROI, mask area (cm^2^) on ROI, mask volume (mL) on ROI from T1CE and T2FLAIR) were separately investigated, obtaining segmental volumes of contrast-enhancing tumor region and edematous or infiltrative peritumoral T2FLAIR region, and other relevant volumetric parameters. Overall, 82 volumetric and physiologic parameters were investigated in this study (Table 2).

**Table 2:** Demographic data of 65 glioblastomas

	Total (n=65)
Age (years)	57.7 (29.0-74.0)
Gender (male)	35 (53.8)
IDH	
IDH-mutant	5 (7.7)
IDH-wild type	60 (92.3)
*MGMT* status methylated/unmethylated	24/ 41
KPS (%)	89.0 ± 14.3
Overall survival (months)	13.2(95% CI 9.3-17.6)

Values are number (%), median (range), or mean ± SD.

### Tissue specimens

Following receipt of informed consent in accordance with the appropriate IRB, glioblastoma specimens were obtained from patients undergoing surgery. For genomic analysis, tumor specimens that were diagnosed by the pathologists were snap-frozen and preserved in liquid nitrogen. Genomic DNA and mRNA were extracted using the DNeasy kit and the RNeasy kit (Qiagen), respectively for whole exome and transcriptomic sequencing.

### Next-generation RNA sequencing (RNA-Seq)

RNA-Seq was performed for all 65 patients. RNA-Seq–based transcriptome profiling was performed by the Samsung Institute for Intractable Cancer Research (Seoul, Korea) using the Illumina TrueSeq RNA Sample Prep kit. Trimmed sequenced reads of 30 nucleotides (nt) were mapped on hg19 using GSNAP and the resulting alignment SAM files were summarized into BED files using SAMtools and bedTools (bamToBed). DEGseq R package was used to calculate RPKM (Reads Per Kilobase of transcript per Million reads) values from the hg19 refFlat file downloaded from the UCSC genome browser and the BED files during the per nucleotide coverage analysis. Log transformation was applied to correct for the skewed distribution.

### Somatic mutation

The sequenced reads in FASTQ files were aligned to the human genome assembly (hg19) with Burrows-Wheeler Aligner version 0.6.2. Before mutation calling using SAMtools, Picard version 1.73, and Genome Analysis ToolKit (GATK version 2.5.2.), the initial alignment BAM files were subjected to conventional preprocessing We used MuTect (version 1.1.4), SomaticIndelDetector (GATK version 2.2) and Variant Effect Predictor (VEP).

### Copy number variation

The ngCGH python package was used to generate aCGH-like data from whole exome sequencing (WES). Matching normal samples were used as the reference for calculating copy number variations in tumors. Segmentation and copy number calculation of each gene were performed.

### Data analysis

Unsupervised hierarchical clustering was performed on the radiomic phenotypes extracted from the quantitative MR imaging data using NordicICE software program. Before clustering analysis, all radiomic phenotypes were standardized to have a zero mean and a unit standard deviation for clustering analysis and producing heatmap. We then performed consensus clustering (Pearson’s correlation coefficients) on the radiomic phenotypes. We selected k = 3 as it gives sufficiently stable similarity matrix. Putative candidates of which radiomic phenotypes correlated to mRNA gene expression were identified to determine subtypes or clusters that are driven by different mechanisms. This was done using Mann Whitney *U*-test with p<0.05, and Spearman Correlation Coefficient test with Rho >0.6.
